# Supplementary material for: Analysis of the Sensitivity and Specificity of Histopathological Findings for Diagnosing Lupus Nephritis
Source: Diagnostics (Basel). 2024 Nov 27;14(23):2681. doi: 10.3390/diagnostics14232681 (PMC11640231; doi:10.3390/diagnostics14232681)
Supplement: Supplementary file 1 [file diagnostics-14-02681-s001.zip › diagnostics-3309204-supplementary.pdf]

Supplementary Materials

Analysis of the Sensitivity and Specificity of Histopathological Findings for Diagnosing Lupus Nephritis

Epitácio Rafael da Luz Neto, Maria Brandão Tavares, Ana Gabriela de Jesus Torres de Melo, Washington Luiz Conrado dos Santos, Luís Yu

Tables and Figures:

Table S1. Histologic classification - HCFMUSP

	Lupus nephritis (n 269)
**Histologic class (%)**	
Class II	8 (3.0)
Class III	46 (17.1)
Class IV	104 (38.7)
Class V	46 (17.1)
Class III + V	31 (11.5)
Class IV + V	27 (10.0)
Undetermined	7 (2.6)

HCFMUSP, Hospital das Clinicas da Faculdade de Medicina da Universidade de São Paulo

Table S2. Etiological distribution - HCFMUSP

	Control (n 219)
**Histopathologic diagnostic (%)**	
IgA nephropathy	88 (40.2)
Membranous nephropathy	47 (21.5)
Membranoproliferative glomerulonephritis	31 (14.2)
Pauci-imune glomerulonephritis	32 (14.6)
Proliferative glomerulonephritis	21 (9.6)

HCFMUSP, Hospital das Clinicas da Faculdade de Medicina da Universidade de São Paulo

Table S3. Characterization of histopathologic findings in optic microscopy - HCFMUSP

	Lupus nephritis (n 269)	Control (n 219)	IgA nephropathy (n 88)	MN (n 47)	MPGN (n 31)	Pauci-immune GN (n 32)	Proliferative GN (n 21)
Mesangial proliferation	237 (88.8)	140 (63.9)	67 (76.1)	19 (40.4)	31 (100)	9 (28.1)	14 (66.7)
Endocapillary hypercellularity	171 (64.0)	80 (36.5)	30 (34.1)	8 (17.0)	23 (74.2)	6 (18.7)	13 (61.9)
Cellular or fibrocellular crescents							
Absent	123 (46.2)	145 (66.2)	65 (73.9)	45 (95.7)	21 (67.8)	2 (6.2)	12 (57.1)
Less than 25%	77 (29.0)	36 (16.4)	17 (19.3)	2 (4.3)	9 (29.0)	7 (21.9)	1 (4.8)
25 a 50%	46 (17.3)	16 (7.3)	4 (4.5)	0	0	9 (28.1)	3 (14.3)
Greater than 50%	20 (7.5)	22 (10.1)	2 (2.3)	0	1 (3.2)	14 (43.8)	5 (23.8)
Global glomeruloesclerosis							
Less than 25%	214 (81.1)	144 (69.2)	51 (61.4)	37 (84.1)	24 (77.4)	17 (54.8)	15 (79.0)
25 a 50%	33 (12.5)	41 (19.7)	17 (20.5)	6 (13.6)	6 (19.4)	10 (32.3)	2 (10.5)
Greater than 50%	17 (6.4)	23 (11.1)	15 (18.1)	1 (2.3)	1 (3.2)	4 (12.9)	2 (10.5)
Interstitial fibrosis/Tubular atrophy							
Less than 25%	228 (85.7)	168 (76.7)	62 (70.5)	35 (74.5)	26 (83.9)	26 (81.3)	19 (90.5)
25 a 50%	28 (10.5)	40 (18.3)	19 (21.6)	10 (21.3)	4 (12.9)	5 (15.6)	2 (9.5)
Greater than 50%	10 (3.8)	11 (5.0)	7 (7.9)	2 (4.2)	1 (3.2)	1 (3.1)	0
IF/TA or global GS > 50%	30 (11.4)	38 (18.1)	20 (23.5)	5 (11.4)	3 (9.7)	7 (22.6)	3 (15.8)
Acute vascular changes	39 (14.5)	28 (12.8)	13 (14.8)	1 (2.2)	8 (25.8)	6 (18.7)	0
Chronic vascular changes	182 (67.7)	163 (74.4)	66 (75.0)	38 (80.1)	25 (80.1)	20 (62.5)	14 (66.7)

Note: Data are presented as n (%). Missing data were excluded from the analysis. HCFMUSP, Hospital das Clinicas da Faculdade de Medicina da Universidade de São Paulo; MN, membranous nephropathy; MPGN, membranoproliferative glomerulonephritis; IF/TA, intersticial fibrosis/tubular atrophy; GS, glomerulosclerosis

Table S4. Combined diagnostic performance of histopathological features in distinguishing class V lupus nephritis and non-lupus membranous nephropathy - HCFMUSP

Nº of features*	Sensitivity (IC 95%)	Specificity (IC 95%)	Accuracy (IC 95%)	PPV (IC 95%)	NPV (IC 95%)	Positive LR (IC 95%)	Negative LR (IC 95%)
One or more	0.91 (0.79, 0.98)	0.40 (0.26, 0.56)	0.66 (0.55, 0.76)	0.61 (0.48, 0.72)	0.82 (0.60, 0.95)	1.52 (1.18, 1.96)	0.22 (0.08, 0.59)
Two or more	0.76 (0.61, 0.87)	0.89 (0.76, 0.96)	0.82 (0.73, 0.90)	0.88 (0.73, 0.96)	0.78 (0.65, 0.89)	6.85 (2.95, 15.90)	0.27 (0.16, 0.46)
Three	0.46 (0.31, 0.61)	0.98 (0.88, 1.00)	0.71 (0.61, 0.80)	0.95 (0.77, 1.00)	0.64 (0.51, 0.75)	20.54 (2.88, 146.36)	0.56 (0.42, 0.73)

**Mesangial deposits, positive C1q and 4 or more positive elements in immunofluorescence.* HCFMUSP, Hospital das Clinicas da Faculdade de Medicina da Universidade de São Paulo; PPV, positive predictive value; NPV, negative predictive value; LR, likelihood ratio;

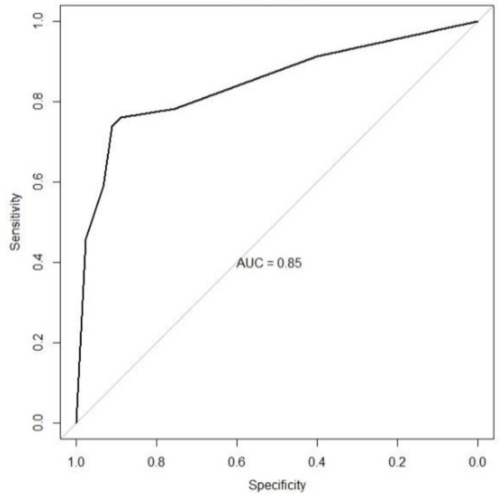

Figure S1. ROC curve to distinguish class V lupus nephritis and non-lupus membranous nephropathy - HCFMUSP

**Table S5. Demographic and clinical characteristics - HAN patients**			
	Lupus Nephritis (n 269)	Control (n 219)	
Demographic data:			
Age	29 (21-37)	35 (24-45)	**p=0.003**
Sex			**p<0.001**
Male	28 (19.1)	48 (60.8)	
Female	136 (82.9)	31 (39.2)	
Race/Ethnicity			**p<0.001**
Black	62 (37.8)	15 (19.0)	
Mixed	45 (27.4)	10 (12.7)	
White	14 (8.6)	9 (11.4)	
Yellow	0	0	
Unknown	43 (26.2)	45 (56.9)	
Clinical data:			
Creatinine (mg/dl):	1,33 (0,80-2,52)	1,27 (1,0-2,45)	p=0.37
eGFR (CKD-EPI):	59 (25 - 98)	65 (26-86)	p=0.97
Hemoglobin (g/dl):	9,4 (7,9-11,5)	12,1 (10,2-13,5)	**p<0.001**
Albumin (g/dl):	2,3 (2,0-2,9)	3,0 (2,4-3,6)	**p<0.001**
Cholesterol (mg/dl):	219 (180-277)	225 (175-285)	p=0.89
Proteinuria (g/d or g/g):	4,624 (2,037-7,862)	3,687 (1,675-8,220)	p=0.43
Hematuria (%):	124 (82.1)	47 (66.2)	**p=0.008**
Positive ANA (%):	142 (98.6)	7 (14.6)	**p<0.001**
Renal syndrome (%):			p=0.29
Acute glomerulonephritis	7 (4.3)	4 (6.1)	
Nephrotic syndrome	47 (29.0)	20 (30.3)	
Nephritic/nephrotic syndrome	50 (30.9)	13 (19.7)	
Rapidly progressive GN	15 (9.3)	4 (6.1)	
Unknown etiology renal injury	8 (4.9)	7 (10.6)	
Asymptomatic urinary abnormalities	35 (21.6)	18 (27.2)	

All data are displayed as median (interquartile range) or N (%). HCFMUSP, Hospital das Clinicas da Faculdade de Medicina da Universidade de São Paulo; eGFR, estimated glomerular filtration rate; CKD-EPI, Chronic Kidney Disease Epidemiology Collaboration; ANA, anti-nuclear antibody; GN, glomerulonephritis

**Table S6. Histologic classification - HAN**	
	Lupus nephritis (n 164)
**Histologic class (%)**	
Class II	1 (0.6)
Class III	16 (9.8)
Class IV	75 (45.7)
Class V	38 (23.2)
Class III + V	14 (8.5)
Class IV + V	16 (9.8)
Undetermined	4 (2.4)

HAN, Hospital Ana Nery

**Table S7. Etiological distribution - HAN**	
	Control (n 79)
**Histopathologic diagnostic (%)**	
IgA nephropathy	22 (27.8)
Membranous nephropathy	28 (35.4)
Membranoproliferative glomerulonephritis	7 (8.9)
Pauci-imune glomerulonephritis	4 (5.1)
Proliferative glomerulonephritis	18 (22.8)

HAN, Hospital Ana Nery
